# Gastroduodenal Artery Pseudoaneurysm: A Rare Cause of Upper Gastrointestinal Bleeding and Pancreatic Duct Compression

**DOI:** 10.7759/cureus.29971

**Published:** 2022-10-06

**Authors:** Ahmad S Alam, Mostafa Elkhawaga, Kanica Yashi

**Affiliations:** 1 General Surgery, John Hunter Hospital, Newcastle, AUS; 2 Internal Medicine, Bassett Healthcare Network, Cooperstown, USA

**Keywords:** visceral artery pseudoaneurysms, angioembolization, gastroduodenal artery, acute gastrointestinal bleed, complications of pancreatitis

## Abstract

Visceral artery pseudoaneurysm as a cause of upper gastrointestinal bleeding is a rare occurrence. These pseudoaneurysms occur most commonly in the splenic artery but have been reported in the gastroduodenal artery as well, albeit with a high mortality rate in cases of rupture. We present a case of a gastroduodenal pseudoaneurysm presenting with upper gastrointestinal bleeding and causing a mass effect on the pancreatic duct as well.

## Introduction

Pseudoaneurysms are potentially life-threatening lesions that have been reported in almost all major visceral arteries [1]. It commonly develops secondary to inflammation or trauma [2]. They present as a hematoma usually after destruction of the vascular wall, unlike a true aneurysm which has all three layers of an artery [3]. This puts pseudoaneurysms at a higher risk of rupture than true aneurysms, which has also been demonstrated in studies [4].

## Case presentation

An 84-year-old female presented with a four-week history of black stools which was also associated with intermittent epigastric pain. She reported experiencing a similar epigastric pain a few hours ago which she described as sharp, 7/10 in intensity, radiating to the back but had self-resolved in two hours. The patient also complained of some episodes of dizziness in the last few days, which prompted her presentation to the emergency department. Her past medical history was significant for hypertension, which was well controlled. Her past surgical history was indicative of cholecystectomy and a right hip replacement.

On examination, the patient was hemodynamically stable, with a blood pressure of 124/84 mmHg and a heart rate of 80 beats per min. Her temperature was noted to be 36.6°C and she was saturating at 98% on room air. Her abdominal examination was unremarkable with mild tenderness in the epigastrium, but no signs of peritonism. A digital rectal examination did not reveal evidence of melena.

She underwent a series of laboratory tests that have been detailed in Table 1. It showed decreased hemoglobin with a microcytic hypochromic picture on analysis. The other significant blood test result was a raised lipase and an increased c-reactive protein. Her liver function tests and kidney function tests were unremarkable and within normal limits. These have been compared to her baseline results from two months ago.

**Table 1:** Laboratory investigations comparing blood tests at the time of presentation and patients baseline test results from two months ago.

Laboratory investigations	Results	Baseline results	Normal range
Hemoglobin	77 g/L	96 g/L	115-165 g/L
Lipase	1400 U/L	-	10-60 U/L
Bilirubin	6 umol/L	7 umol/L	<20 umol/L
Creatinine	78 umol/L	72 umo/L	45-90 umol/L
C-reactive protein	116 mg/L	-	<5 mg/L

**Figure 1:**
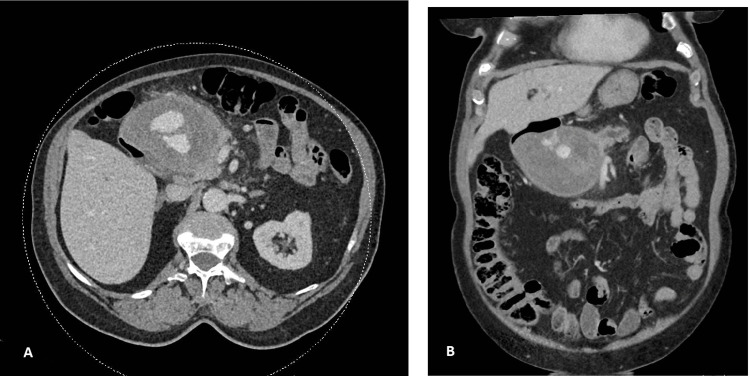
Computed tomography showing axial (A) and coronal (B) images of gastroduodenal artery pseudoaneurysm measuring 84×99×76 mm.

**Figure 2:**
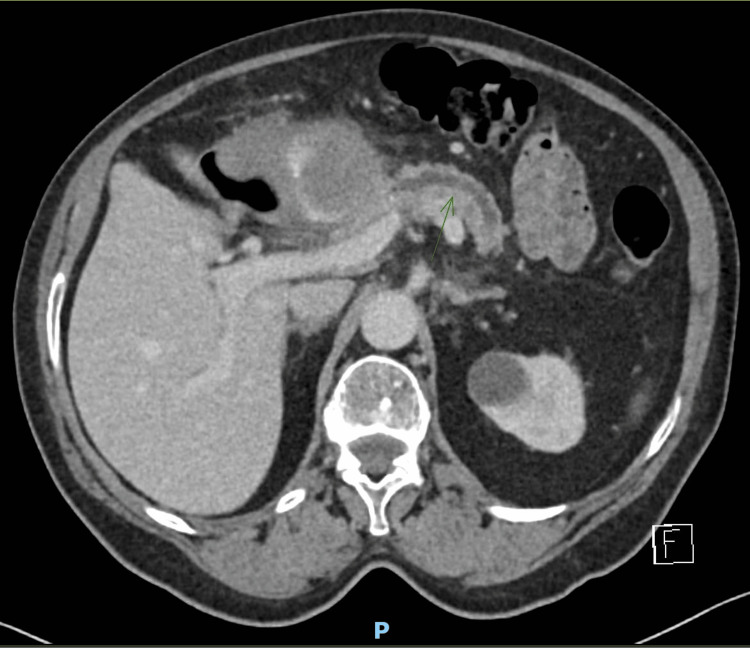
Computed tomography showing an axial image of gastroduodenal artery pseudoaneurysm causing pancreatic duct obstruction.

She underwent a CT scan as per advice from the gastroenterology consultant, which revealed a large (84x99x76 mm) mass lesion suggestive of a gastroduodenal pseudoaneurysm (Figures 1A, 1B). The lesion was also causing a mass effect on the pancreatic duct (Figure 2).

**Figure 3:**
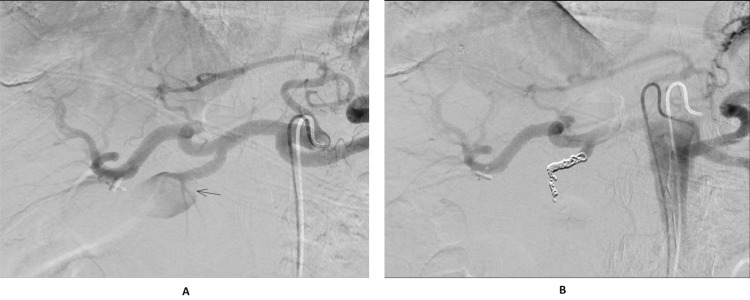
Angiographic images showing pseudoaneurysm of gastroduodenal artery (A) and completion angiography showing successful coiling of pseudoaneurysm with preserved collateral flow (B).

The interventional radiology team was consulted who reviewed the images and booked the patient in for a procedure the next morning. She underwent angiographic coil embolization of the gastroduodenal artery without post-procedural complications (Figures 3A, 3B). Her recovery in the ward was uneventful, and she was discharged with a plan to follow up in two weeks. Our patient had a repeat CT scan after two weeks which showed a stable collection and no active bleeding. She had another CT scan in three months which showed significant resolution of the collection from 10 cm to 3.8 cm. The patient was asymptomatic for both of the consultations.

## Discussion

In cases of gastroduodenal artery pseudoaneurysms, which make up about 1.5% of reported visceral pseudoaneurysms, the most common cause is usually proteolytic destruction of wall by pancreatic enzymes released secondary to chronic pancreatitis [5]. Studies have shown the incidence of pseudoaneurysms from pancreatitis to be around 1.3-17% [5,6]. Overall, the detection of such lesions incidentally has increased in asymptomatic patients due to a rise in use and availability of imaging studies, such as computerized tomography scans, magnetic resonance imaging, and ultrasonography.

The most common presentation noted in studies for pseudoaneurysms is gastrointestinal bleeding followed by abdominal pain [7]. Both of these symptoms were present in our patient. Apart from this, there are also reports of patients presenting with obstructive jaundice due to extrinsic compression of the bile ducts [8].

Although the gold standard imaging for diagnosis of pseudoaneurysms is angiography [7], a CT scan is the most commonly used and also the most sensitive of all the non-invasive modalities of imaging [9]. Ultrasonography has a lower sensitivity but is effective as an initial screening tool. In cases of gastroduodenal artery pseudoaneurysms, it is essential to assess the images in multiplanar reformations as it can easily be confused with soft tissue masses of the pancreatic head or duodenal masses.

Over the past decade, an endovascular approach to managing stable patients with pseudoaneurysms has become the first line, with open vascular repair reserved for hemodynamically unstable patients or in those who have failed endovascular approaches [10]. Endovascular embolization of such lesions has success rate of around 94% in a series [11] and has been employed successfully for 100% of cases in another [12]. As there is no correlation between the size of the pseudoaneurysm and its chance of rupture, asymptomatic patients are also routinely embolized to prevent mortality if untreated, which is as high as 90% for a gastroduodenal artery (GDA) pseudoaneurysm [2]. Patients do need to be made aware that transcatheter embolization is associated with risks of ischemia and infarction of organs, coil migration, and contrast-induced nephropathy [13].

## Conclusions

In conclusion, it is pertinent for clinicians to consider a visceral artery pseudoaneurysm as a cause of upper gastrointestinal bleeding, especially in the setting of acute or chronic pancreatitis. A timely diagnosis could prevent a fatal outcome in such patients through appropriate endovascular or surgical repair.
